# Appraisement of aerobic capacity among first medical students following a regular yogic regime

**DOI:** 10.6026/9732063002001271

**Published:** 2024-10-31

**Authors:** Ruchi Kothari, Yogesh S, Mayur Wanjari, Labdhi Sangoi, Ravi Sangoi

**Affiliations:** 1 Department of Physiology, Mahatma Gandhi Institute of Medical Sciences, Sevagram, Wardha, Maharashtra, India; 2 Department of Internal Medicine, Institute of Internal Medicine, Madras Medical College and Rajiv Gandhi Government General Hospital, Chennai, India; 3 MHPE Scholar, Department of Internal Medicine Sri Balaji Vidyapeeth University, Pillaiyarkuppam, Pondicherry, Tamilnadu, India; 4 Department of Research, Datta Meghe Institute of Higher Education & Research (DMIHER), Sawangi, Maharashtra, India; 5 Department of Research, Government Medical College, Jalna, Maharashtra; 6 Department of Internal Medicine, Government Medical College and General Hospital, Baramati, Pune, Maharashtra, India

**Keywords:** Aerobic capacity, academic load, cardiorespiratory, stress factor, yoga, exercise

## Abstract

The heavy academic load among medical students can be a stressful factor affecting not only the physical fitness of the medical student but also his or her mental well-being. Since time immemorial, yogic exercises have been found to decrease both physical and psychological stresses and increase cardiorespiratory fitness. Therefore, it is of interest to assess the effect of a yogic routine on cardiopulmonary efficiency and aerobic fitness as measured by treadmill performance in first-year MBBS students. A prospective interventional study involving 100 MBBS students within the age group of 17 to 20 years was conducted. In each participant, the baseline cardiopulmonary parameters, VO2 max, were recorded before and after two months of intervention with yoga (1 hour/day). SPSS was used to perform the statistical analysis. There was a significant improvement in VO2 max (35.4 ± 5.2 to 40.1 ± 4.8 ml/kg/min, p < 0.001) and rest heart rate (75.3 ± 8.1 to 68.5 ± 6.9 beats/min, p < 0.001). Practice of yoga regularly improves the aerobic capacity and cardiopulmonary efficiency of the student who studies MBBS. Yoga may help students better their well-being and academic performance if practiced as part of the MBBS curriculum.

## Background:

Transitioning to medical college is stressful academically, which adversely affects the physical and mental health of students. First-year MBBS students are usually prone to stress, anxiety and depression, thereby hampering academics and well-being [1]. Yoga, which includes postures, breathing practices and meditation, has been shown to improve physical fitness and mental health. Studies in the past have revealed that it enhances great health measures like cardiovascular and respiratory functions, whereas it decreases psychological distress [2]. This study was designed to assess the impact of a routine yogic schedule on cardiopulmonary efficiency with regard to VO2 max, which happens to be one of the most important markers of aerobic fitness. The idea is to compare the pre- and post-yoga intervention values to find out if yoga can improve the condition of physical health and help to relieve stress among the first-year MBBS students [3].

## Methodology:

This is a two-month interventional study done at Mahatma Gandhi Institute of Medical Sciences, Sevagram, from June 10 to August 10, 2022. A hundred first-year MBBS students aged 17-20 years were enrolled. Those students with debilitating diseases or on medications that affect autonomic reflexes were excluded.

## Intervention:

Students participated in daily 1-hour yoga sessions, including warm-ups, postures, pranayama (breathing exercises) and relaxation/meditation techniques. The sessions were led by a certified yoga instructor from the Arogyadham center.

## Post-Intervention evaluation:

After two months, the same cardiopulmonary and psychological assessments were repeated. Data was analyzed using SPSS version 23. Paired t-tests were used to assess differences between pre- and post-intervention values, with p < 0.001 considered statistically significant. Subgroup analyses by gender, BMI and age groups were also conducted.

## Ethical considerations:

This study was approved by the Institutional Ethics Committee of Mahatma Gandhi Institute of Medical Sciences. Informed consent was obtained from all participants [12]. Participants were informed about the study's purpose, procedures, benefits, and risks, and they were free to withdraw at any point.

## Results:

Table 1 shows demographic Characteristics of the Study Population. Table 2 shows baseline cardiopulmonary parameters. Table 3 shows post-Intervention cardiopulmonary parameters. Table 4 shows comparison of pre- and post-intervention parameters. Table 5 shows changes in VO2 Max by Gender. Table 6 shows the impact of BMI on VO2 Max Improvement. Table 7 shows change in resting heart rate by age group. Table 8 shows comparison of respiratory parameters. Table 9 shows effect of yoga on perceived stress levels. Table 10 shows improvement in academic performance of the students. Table 11 shows adherence and acceptability of the yoga program. Table 12 shows adverse events and discomfort during the regime.

The intervention led to significant improvements in cardiorespiratory parameters. The mean VO2 max increased from 35.4 ± 5.2 to 40.1 ± 4.8 ml/kg/min (p < 0.001), while the resting heart rate decreased from 75.3 ± 8.1 to 68.5 ± 6.9 beats/min (p < 0.001). Blood pressure and respiratory rate also improved significantly. Psychological measures showed a reduction in perceived stress, anxiety and depression scores. The average age of participants was 18.5 years, with a balanced distribution of males and females. The mean BMI indicates that most participants were within the normal weight range. Baseline measurements show that participants had an average VO2 max of 35.4 ml/kg/min, indicating moderate cardiorespiratory fitness. Post-intervention data show significant improvements in all measured parameters, with a notable increase in VO2 max and a decrease in resting heart rate. The results indicate a significant improvement in all measured cardiopulmonary parameters following the yoga intervention, with a marked increase in VO2 max and reductions in heart rate, blood pressure, and respiratory rate. Both male and female students showed significant improvements in VO2 max, with males having a slightly higher baseline and post-intervention VO2 max. However, the improvement was significant in both genders.

All BMI categories showed significant improvements in VO2 max, suggesting that the benefits of yoga are consistent across different body compositions. Both age groups showed significant reductions in resting heart rate, indicating improved cardiovascular efficiency. Significant improvements were observed in respiratory parameters such as tidal volume, peak expiratory flow and forced vital capacity following the yoga intervention, indicating enhanced respiratory efficiency. There was a significant reduction in perceived stress, anxiety, and depression scores among the students, post-intervention, suggesting a positive mental health impact of regular yoga practice. Regular yoga practice not only improved physical and mental health but also positively impacted academic performance, as evidenced by higher concentration levels, increased study hours and improved examination scores. Most of the participants adhered to the yoga program, reported high levels of satisfaction, and expressed willingness to continue practicing yoga, highlighting the acceptability and perceived benefits of the intervention. The incidence of adverse events was minimal, with most participants reporting no adverse effects, indicating the safety of the yoga intervention.

## Discussion:

The cardiopulmonary parameters, aerobic capacity and mental well-being of the first-year MBBS students significantly improved following the regular yogic regime in this study [4]. VO2 max was improved maximally, followed by resting heart rate and respiratory parameters. Hence, cardiopulmonary efficiency was improved. Results were consistent with the studies that indicated that yoga enhanced physical fitness in the form of better oxygen utilization and reduced levels of stress [5]. The levels of perceived stress, anxiety and depression were also significantly influenced by yoga. Medical students experience great pressure when studying medicine, damaging both their academic performance and well-being [6]. The present study highlights the prospect of yoga for students as a viable stress management strategy, especially in a high-pressure environment [7]. The improvement of academic scores suggests that practice in the yogic tradition aids in the development of greater concentration, memory, and efficiency in learning. Such enhancement may possibly be because these improvements enhance brain functions with yoga by promoting clarity in the mind and assisting in maintaining the focus of the mind [8]. Adherence to the yoga program was high, with most of the participants attending sessions above 90% and satisfactions for the intervention. Minimal adverse events incidence further supports the safety and feasibility of incorporating yoga into the medical curriculum [9].

Although this study has promising results, several limitations exist in this study. A short study period as well as single-centre design might limit the generalizability of findings [10]. The study also relied on self-reported measures of stress and academic performance, which are generally open to bias [11]. Future studies should use objective measures of stress by using cortisol levels and cognitive assessment in terms of academic performance. A more extended follow-up would be useful for assessing the long-term effects of yoga on both physical and mental health outcomes [12]. The gender and BMI subgroup analyses from this study tell an important story about the differential effects of yoga. While boys and girls, as well as students of all different BMI categories showed considerable improvement in VO2 max and other parameters, the practice was shown to be helpful regardless of gender or body composition. This lends testimony to the adaptability of yoga as an intervention for a diversely composed student population [13]. The marked reduction in self-rated stress and betterment of indices of mental health are particularly impressive. In the wake of increasing prevalence of mental health issues among medical students, yoga may be introduced in colleges as a preventive tool against burnout and psychological distress. Universities could consider providing formal access to yoga classes as part of student wellness programs [14]. It would improve academic performance, as indicated through higher concentration levels and examination scores, suggesting academic benefit. With enhanced focus and diminished stress, there is more potential for great learning outcomes and better retention of complex medical knowledge [15]. A high adherence rate and interest to continue with the practice among participants showed acceptability and feasibility of integrating yoga into the schedules of busy medical students. Critical for long-term success and sustainability of the programmes, institutes should be able to find several flexible arrangements of yoga modules that fit with the tastes and requirements of the students to ensure continued engagement.

## Conclusion:

Regular yoga practice significantly improves aerobic capacity, cardiopulmonary efficiency, and mental well-being in first-year MBBS students. The findings of this study suggest that yoga can be an effective tool for enhancing physical fitness and reducing stress among medical students, thereby promoting a healthier and more conducive learning environment. Given its wide range of benefits and minimal risk of adverse events, yoga should be considered as an integral part of the medical education curriculum. Future research should focus on multi-institutional studies with larger sample sizes and extended follow-up periods to confirm these findings and explore the long-term impact of yoga on student health and academic success.

## Figures and Tables

**Table 1:** Demographic characteristics of the study population

**Variable**	**Mean ± SD**
Age (years)	18.5 ± 1.1
Gender (M/F)	55/45
**BMI (kg/m^2^)**	22.8 ± 3.4

**Table 2:** Baseline cardiopulmonary parameters

**Parameter**	**Mean ± SD**
Resting Heart Rate (bpm)	75.3 ± 8.1
Systolic Blood Pressure (mmHg)	118.4 ± 10.5
Diastolic Blood Pressure (mmHg)	76.2 ± 7.8
Respiratory Rate (breaths/min)	16.8 ± 2.3
VO2 Max (ml/kg/min)	35.4 ± 5.2

**Table 3:** Post-Intervention cardiopulmonary parameters

**Parameter**	**Mean ± SD**
Resting Heart Rate (bpm)	68.5 ± 6.9
Systolic Blood Pressure (mmHg)	112.5 ± 9.8
Diastolic Blood Pressure (mmHg)	73.5 ± 6.5
Respiratory Rate (breaths/min)	15.2 ± 2.0
VO2 Max (ml/kg/min)	40.1 ± 4.8

**Table 4:** Comparison of pre- and post-intervention parameters

**Parameter**	**Baseline Mean ± SD**	**Post-Intervention Mean ± SD**	**p-value**
Resting Heart Rate (bpm)	75.3 ± 8.1	68.5 ± 6.9	<0.001*
Systolic Blood Pressure (mmHg)	118.4 ± 10.5	112.5 ± 9.8	<0.001*
Diastolic Blood Pressure (mmHg)	76.2 ± 7.8	73.5 ± 6.5	<0.001*
Respiratory Rate (breaths/min)	16.8 ± 2.3	15.2 ± 2.0	<0.001*
VO2 Max (ml/kg/min)	35.4 ± 5.2	40.1 ± 4.8	<0.001*

**Table 5:** Changes in VO2 Max by Gender

**Gender**	**Baseline VO2 Max (ml/kg/min)**	**Post-Intervention VO2 Max (ml/kg/min)**	**p-value**
Male	36.2 ± 5.5	41.0 ± 5.0	<0.001*
Female	34.3 ± 4.8	39.0 ± 4.6	<0.001*

**Table 6:** Impact of BMI on VO2 Max Improvement

**BMI Category**	**Baseline VO2 Max (ml/kg/min)**	**Post-Intervention VO2 Max (ml/kg/min)**	**p-value**
Underweight (<18.5)	34.0 ± 4.2	38.5 ± 4.0	< 0.001*
Normal (18.5-24.9)	35.8 ± 5.0	40.3 ± 4.7	<0.001*
Overweight (> 25)	36.5 ± 5.8	40.7 ± 5.1	<0.001*

**Table 7:** Change in resting heart rate by age group

**Age Group (years)**	**Baseline Resting \Heart Rate (bpm)**	**Post-Intervention Resting Heart Rate (bpm)**	**p-value**
17-18	74.8 ± 7.5	68.0 ± 6.5	<0.001*
19-20	75.9 ± 8.8	69.2 ± 7.3	<0.001*

**Table 8:** Comparison of respiratory parameters

**Parameter**	**Baseline Mean ± SD**	**Post-Intervention Mean ± SD**	**p-value**
Respiratory Rate (breaths/min)	16.8 ± 2.3	15.2 ± 2.0	< 0.001*
Tidal Volume (ml)	500.0 ± 75.0	550.0 ± 80.0	< 0.001*
Minute Ventilation (L/min)	8.4 ± 1.8	8.3 ± 1.6	0.04*
Peak Expiratory Flow (L/min)	350.0 ± 55.0	380.0 ± 60.0	< 0.001*
Forced Vital Capacity (L)	4.2 ± 0.8	4.5 ± 0.9	< 0.001*

**Table 9:** Effect of yoga on perceived stress levels

**Parameter**	**Baseline Mean ± SD**	**Post-Intervention Mean ± SD**	**p-value**
Perceived Stress Score	20.5 ± 6.4	15.2 ± 5.1	< 0.001*
Anxiety Score	12.8 ± 4.5	8.7 ± 3.9	< 0.001*
Depression Score	10.3 ± 4.0	6.9 ± 3.5	< 0.001*

**Table 10:** Improvement in academic performance

**Academic Parameter**	**Baseline Mean ± SD**	**Post-Intervention Mean ± SD**	**p-value**
Concentration Levels (1-10 scale)	6.5 ± 1.8	8.0 ± 1.5	<0.001*
Study Hours per Day	5.0 ± 1.5	6.5 ± 1.2	<0.001*
Examination Scores (%)	65.0 ± 10.5	72.0 ± 9.8	<0.001*

**Table 11:** Adherence and acceptability of the yoga program

**Parameter**	**Percentage (%)**
Regular Attendance (≥ 90% sessions)	85
Reported Satisfaction with Program	90
Willingness to Continue Yoga	80
Reported Benefits (Physical/Mental)	95

**Table 12:** Adverse events and discomfort

**Adverse Event**	**Number of Cases (n=100)**	**Percentage (%)**
Muscle Soreness	5	5
Dizziness during Practice	2	2
Respiratory Discomfort	1	1
No Adverse Events Reported	92	92

## References

[1] Miller PM (1994). Med Educ..

[2] Stewart SM (1995). Med Educ..

[3] Supe AN (1998). J Postgrad Med..

[4] Abraham RR (2009). South East Asian J Med Educ..

[5] Srinivasan K (2006). Indian J Physiol Pharmacol..

[6] Gupta N (2006). Indian J Physiol Pharmacol..

[7] Whooley A (2004). Altern Ther Health Med..

[8] Schell FJ (1994). In J Psychosom..

[9] Wood C (1993). JR Soc Med..

[10] Kothari R (2023). Cureus..

[11] Malathi A, Damodaran A (1999). Indian J Physiol Pharmacol..

[12] Mittal G (2023). Cureus..

[13] Harinath K (2004). J Altern Complement Med..

[14] Ankad RB (2011). Heart Views..

[15] Bhavanani AB (2012). Int J Yoga Therap..

